# Annotations

**Published:** 1894-09-15

**Authors:** 


### Annotations.

### The Golf "Cure" for Insomnia.

A writer in the *Scotsman*, commenting upon the Marquis of Salisbury's recent address to the members of the British Association at Oxford, expressed regret that his lordship should be a victim to the modern and very prevalent disorder of insomnia. He went on to appeal to the medical profession for some prompt and effective remedy for that distressing ill. "Would that the noble leader of the Conservative party would take a three months' course of golf" was the inward exclamation of one who had himself suffered from insomnia, and was at the time rejoicing in a succession of nights of profound and refreshing sleep. Golf is the game for the exhausted brain-worker at any stage of his life. No junior is too young, no senior is too old to learn it; to learn it and to enjoy it. The proof of the pudding is in the eating. On the golf links of St. Andrews the man of seventy looks fifty and the man of fifty has the appearance of thirty-five. The chief reasons for this are that both the old man and the middle-aged can eat, digest, and sleep. There are scores, perhaps hundreds, of educated men of all classes, between the ages of forty and ninety, at St. Andrews at this moment, and they are, without exception, the healthiest and handsomest collection of middle-aged and old men the writer has ever seen. Sleeplessness, so far as the writer was able to discover, in a three weeks' sojourn at St. Andrews, is absolutely unknown to the regular golf player. One may almost say it is impossible. Living, as he does, in the open air, and taking several hours of daily exercise without unpleasant fatigue, and with a mind constantly, but not laboriously, interested, he eats well and so the brain is adequately nourished. The blood, too, is thoroughly oxydised, and by the due exercise of all the muscles is made to flow evenly throughout the body without abnormal concentration upon the brain. These are the indispensable conditions of sound and certain sleep, and these conditions are admirably fulfilled by regular and systematic golfing. Golf has the merit of being a real "cure" under reasonable physiological conditions, and with the cure of sleeplessness it brings many other advantages, such as strengthened muscles, a toned-up heart, a vigorous appetite, and sound digestion.

### A Clinical Research Association.

New times bring, not only new men but new methods. The times with doctors are emphatically "new"; so new that many busy practitioners are entirely at sea with regard to the knowledge and methods which prevail at the great centres of progressive medical practice. But the centres of practice are most anxious to diffuse their new knowledge on the widest possible scale. There is a zeal for "University Extension" among scientific practitioners, which is resolved that every doctor in the kingdom shall at least have placed before him the possibility of becoming a Jenner or an Andrew Clark. The newest movement of the medical "University Extensionists" is the "Clinical Research Association." There are two questions about the new association, which every practitioner of medicine will ask: First, is it orthodox; and, secondly, is it practical? It is certainly orthodox, as orthodox as distinguished and trusted names can make it. As guarantee of this it is sufficient to print the names of its ten patrons. They are Drs. Clifford Allbutt, Sir W. H. Broadbent, John Cavafy, J. F. Goodhart, T. H. Green, Hughlings Jackson, S. Wilks, and Sir James Paget, Sir J. M. Humphrey, and Mr. Jonathan Hutchinson. There is no doubt also that the association will render great practical service if the profession will use it practically. Take, for instances, the science of bacteriology and the clinical aspects of diseases of the kidneys as test experiments of practicality. The busy practitioner has not the knowledge, has not the craftsmanship for making himself master of all the scientific and all the useful circumstances of the bacterial causes and relations of diseases. But he needs the knowledge, though he does not himself possess the means of acquiring it. Similarly is he placed with regard to the important pathological facts and physiological significance of certain forms of kidney disease. But with all needful scientific knowledge this Clinical Association will furnish him, and that with the strictest regard for his self-respect, and at the smallest possible cost. These are but samples of what the association will do. It proposes, however, to cover the whole field of practical medical science. Every practitioner, whether general or special, should study the prospectus of the new association. Its secretary is Mr. C. H. Wells, and its laboratory is at 5 Denman Street, S.E.

### School Diphtheria in Towns.

Those members of the International Congress of Hygiene who studied together the question of the increase of diphtheria in towns—an increase which, unfortunately, seems to be established beyond a doubt—agreed with practical unanimity that schools were the centres from which epidemics were most frequently propagated. In our times one does not expect a space of half a-century to be interposed between an evil discovered and an evil removed. On the contrary, the scientific mind has become so far universal that one expects to see improvement following upon discovery with the fleet steps of intelligent reason and willing conviction. Our large elementary schools are known to be centres for the spread of diphtheria in large towns. Well, then, means must be at once devised for ridding them of their dangerous power. The proper first steps to be taken are the holding of conferences composed of sanitarians, school managers, and school teachers. The old method, according to which the sanitarian held up the school manager and teacher to public odium and ridicule as an unscientific bungler, and the manager retorted upon the sanitarian that he was an unpractical discoverer of mare's nests is quite out of date. The day of quarrels is over, and the day of symposiums has taken its place. We hope that the expressed conviction of the scientists of Buda Pesth will result in the speedy bringing together of those whose business it is to conserve the life and health of school children, and in the purging of our school life and school buildings from the deadly taints of diphtherial dangers and possibilities.

